# Employment Support Program in Cooperation With the Public Employment Service for Major Depressive Disorder: A Report of Two Cases With Employer Interviews

**DOI:** 10.7759/cureus.105614

**Published:** 2026-03-21

**Authors:** Kojiro Kawano, Saori Nakazawa, Ayako Takehara, Sachie Tanaka

**Affiliations:** 1 Department of Occupational Therapy, Saitama Prefectural University, Saitama, JPN; 2 Department of Rehabilitation Medicine, Medical Corporation Yuaikai, Tikumaso Mental Hospital, Nagano, JPN; 3 Department of Fundamental Occupational Therapy, Shinshu University School of Health Sciences, Nagano, JPN

**Keywords:** collaboration, depressive disorder, employer interview, occupational therapy, public employment service, supported employment, vocational rehabilitation

## Abstract

People with common mental disorders often take recurrent sick leave and experience difficulty sustaining employment, highlighting the need for practical models linking clinical care to workplace support. The Employment Support Program in Cooperation With the Public Employment Service (ESPCP) is a psychiatric day care-based program delivered in partnership with public employment service officials. It combines an intensive, group-based Work Readiness Program with structured coordination with employers during job entry and after placement. We report two cases of patients with major depressive disorder who completed the ESPCP and achieved successful work outcomes. We also integrate employer perspectives collected via semi-structured interviews. Case 1 involved a woman in her 30s with repeated short-term clerical jobs and job loss related to caregiving demands and depressive relapses. Following a three-month ESPCP phase, she obtained a fixed-term position in in-house cleaning (20 hours/week) and remained employed for 12 months, after which she voluntarily transitioned to full-time employment at another company. Key interventions emphasized relapse prevention, crisis planning, and post-hiring consultations regarding interpersonal issues. Case 2 involved a man in his 40s who left a sales position following symptom exacerbation and hospitalization. He completed two consecutive three-month ESPCP phases to rebuild confidence and strengthen relapse prevention with family psychoeducation. He obtained competitive employment in factory manufacturing, starting at 10 hours/week and progressing stepwise to 40 hours/week, with sustained full-time employment at the two-year follow-up visit. Employers described collaboration with the psychiatric day care team and related employment-support professionals as beneficial for sharing timely information, having a clear consultation window, and enabling flexible work design and unified support approaches. The encountered challenges included balancing confidentiality with the need for operational information and deciding the appropriate frequency and content of communication. These cases suggest that ESPCP functions as a practical bridge between clinical services and workplaces for people with depressive disorders and that structured employer collaboration contributes to sustained employment.

## Introduction

Depressive disorders are associated with recurrent sick leave, repeated work disruptions, and job losses [1,2]. In Japan, the number of employees with disabilities, including psychiatric disabilities, has increased [3]. However, early job turnover is common among people with mental disorders, with reports indicating that a large proportion of employees with mental disorders quit within the first few months of employment [4,5]. Workplace mental health problems are also reflected in national surveys on work-related health and safety [6]. The societal cost of depression in Japan is estimated to be larger than that of other mental disorders [7].

From an implementation perspective, workplace interventions and vocational rehabilitation often require coordination among clinical, vocational, and workplace stakeholders. Barriers include confidentiality concerns, stigma, and limited resources, whereas facilitating factors include leadership support and tailored interventions [8,9]. Systematic reviews of vocational rehabilitation have highlighted that collaboration can be a key determinant of successful implementation [10]. Qualitative work on collaborative return-to-work programs emphasizes the importance of clear roles, timely communication, and shared problem-solving [11].

Supported employment models, such as Individual Placement and Support, have an established evidence base that continues to evolve; however, successful delivery depends on practical mechanisms for collaboration and ongoing support [12,13]. Employers’ and coworkers’ perspectives suggest that workplace understanding, communication pathways, and appropriate accommodations influence sustainable employment [14].

In Japan, psychiatric day care settings have increasingly launched Work Readiness Programs (WRPs) and rework programs, including interdisciplinary interventions that may involve occupational therapy [15]. The Employment Support Program in Cooperation With the Public Employment Service (ESPCP) was developed to integrate the intensive WRP within psychiatric day care with structured collaboration among the Public Employment Security Office (Hello Work), the Employment Support Office, and employers [16]. A subsequent retrospective cohort study examined the factors associated with continued employment among people hired after implementation of the ESPCP [17].

We present two cases (practice-report style) focusing on (1) patient-facing interventions and outcomes and (2) employer perspectives gathered through semi-structured interviews. Both patients provided written informed consent for publication.

The novelty of this report lies not in proposing the ESPCP but in presenting implementable knowledge for coordination among the psychiatric day care team, the Public Employment Security Office, the Employment Support Office, and employers. We describe an operational collaboration approach that covers patient consent for sharing work-relevant functional information; the timing and frequency of employer contact; and function-focused information sharing regarding warning signs, workload tolerance, and agreed-upon response plans. Using this structure, we link two complementary data sources, such as clinical trajectories and employer perspectives, to extract actionable implementation insights.

## Case presentation

Program overview

The ESPCP was delivered through collaboration among the psychiatric day care team (treatment facilities), the Public Employment Security Office (Hello Work), and the Employment Support Office (welfare facilities), with each organization having diverse job responsibilities while working collaboratively. In this report, the term “ESPCP team” refers to these collaborating professionals. The core WRP was a structured weekday program (five days per week, six hours per day) for approximately three months [16]. After program entry and during job placement, the ESPCP team conducted regular meetings and telephone calls with employers to support work adjustment, provide guidance on support strategies, and coordinate problem-solving while respecting confidentiality.

Employer collaboration protocol (operational outline)

The employer collaboration protocol (operational outline) includes three phases. (1) Preemployment: Obtain written consent specifying what employee work-relevant functional information may be shared and confirm contact persons and preferred channels. (2) Job entry (initial one to two weeks): A brief check-in (phone or meeting) to confirm the duties, schedule, supervision, and early warning signs/response steps. (3) Stabilization (thereafter): Scheduled contacts approximately monthly, with additional contact triggered by predefined events (e.g., increased fatigue, interpersonal conflict, errors, attendance concerns, or employer uncertainty). The shared information is symptom-neutral and function-focused (workload tolerance, warning signs, and agreed-upon response plans), balancing confidentiality with operational needs.

**Figure 1:**
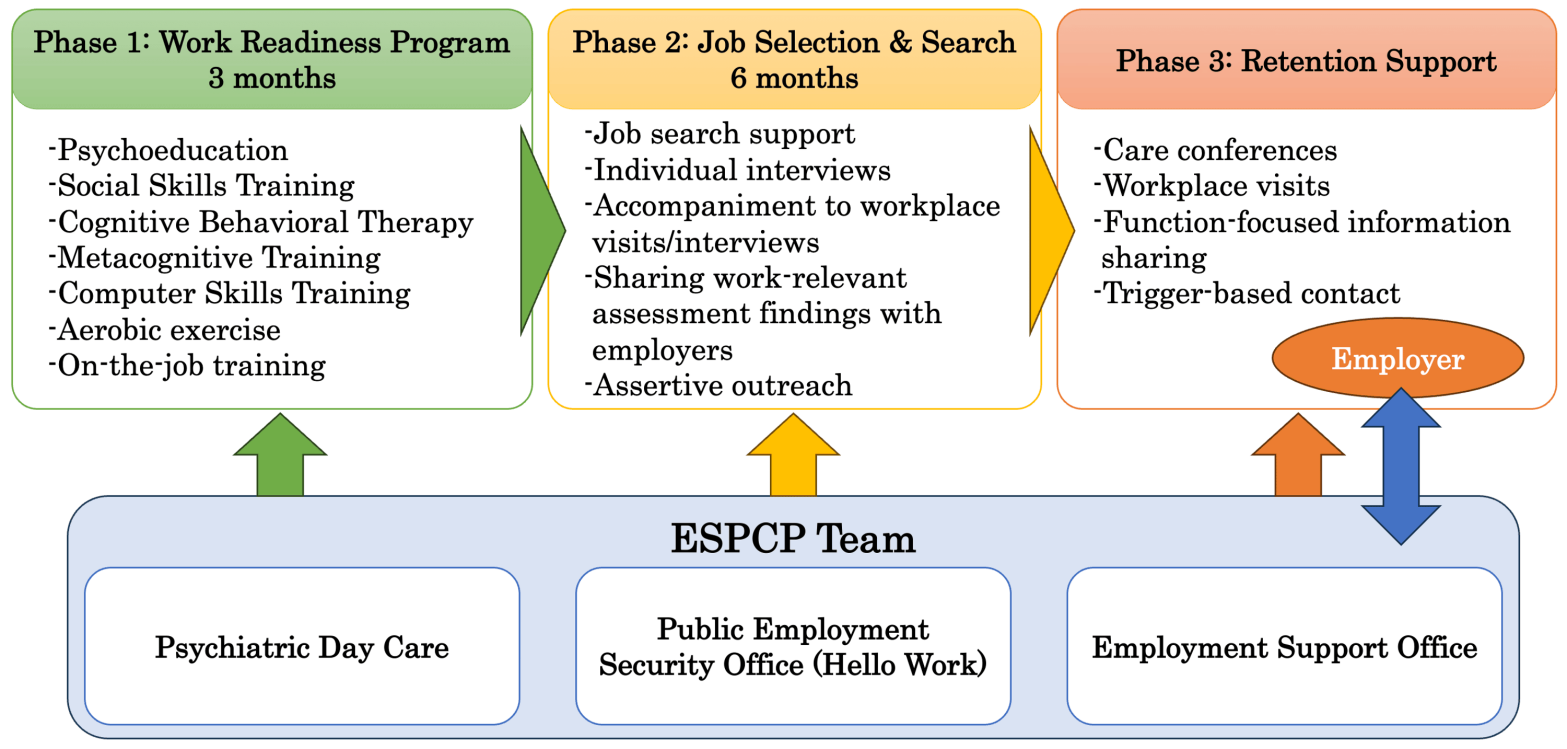
Structure and workflow of ESPCP ESPCP = Employment Support Program in Cooperation With the Public Employment Service This figure was created by the authors using Microsoft PowerPoint (Microsoft Corporation, Redmond, WA, USA).

Figure 1 illustrates the structure of the ESPCP team and the workflow from WRP participation to job retention support, including collaboration with employers during the retention phase.

Outcomes and data collection

The primary outcomes were employment retention (duration of employment at follow-up) and the trajectory of weekly work hours. Secondary outcomes included attendance indicators (sick leave days and late arrivals/early departures), notable work-related difficulties requiring workplace adjustment, and relapse prevention indicators (e.g., the emergence of warning signs and coping responses). Scheduled symptom scales were not routinely administered; instead, global clinical status was tracked using the modified Global Assessment of Functioning (mGAF) [18,19]. Functional changes were tracked using the Life Assessment Scale for the Mentally Ill (LASMI) [20] (D: Daily living; I: Interpersonal relations; W: Work; E: Endurance and stability; R: Self-recognition). This tracking was accompanied by employment and attendance records, clinician documentation, and employer-reported workplace adaptation processes.

A summary of the two cases and key outcomes is provided in Table 1.

**Table 1:** Summary of cases D = daily living; E = endurance and stability; ESPCP = Employment Support Program in Cooperation With the Public Employment Service; I = interpersonal relations; LASMI = Life Assessment Scale for the Mentally Ill; mGAF = modified Global Assessment of Functioning; R = self-recognition; W = work; WRP = Work Readiness Program

Domain	Case 1	Case 2
Diagnosis; demographics (de-identified)	Major depressive disorder; female, 30s	Major depressive disorder; male, 40s
Work history and context	Repeated short-term clerical jobs; job loss linked to caregiving burden and worsening symptoms	Left sales job after symptom exacerbation and hospitalization
ESPCP/WRP duration	One three-month phase	Two consecutive three-month phases
Central WRP elements	Aerobic exercise, psychoeducation, relapse prevention with crisis planning and warning sign monitoring, and post-employment interpersonal consultation	Phase 1: confidence rebuilding and cognitive-behavioral therapy-based cognitive restructuring; Phase 2: relapse prevention and family psychoeducation
Job obtained	In-house cleaning (fixed-term contract)	Factory manufacturing
Outcome	20 hours/week; maintained employment for 12 months; sick leave: one day (menstrual pain); late arrivals/early departures: 0; later transitioned to regular employment at another company by choice	10→15→20→30→40 hours/week (duration: 1 month, 1.5 months, one month, two months, then ongoing); full-time work maintained for two years; sick leave: 0; late arrivals/early departures: 0
mGAF (baseline→end)	68→78	54→61 (after phase 1)→68 (after phase 2)
LASMI domain scores (lower = better)	D: 3→2; I: 10→10; W: 6→3; E: 2→2; R: 6→5	D: 7→4→3; I: 14→14→11; W: 18→11→9; E: 4→4→3; R: 6→6→5

Case 1

A woman in her 30s with major depressive disorder had a history of repeated short-term employment as a clerical worker (three prior companies, each lasting approximately three to six months) and intermittent part-time work in the service industry. She repeatedly left work when caregiving demands at home increased, and her depressive symptoms worsened. She was enrolled in a three-month ESPCP phase. Given her relatively high premorbid cognitive and computer skills and adequate superficial social interaction skills, the WRP emphasized aerobic exercise and psychoeducation.

During the job search and entry phases, the team supported relapse prevention and created a written crisis plan specifying early warning signs, coping strategies, and escalation steps. The Public Employment Security Office helped with job selection and served as a bridge at the time of job placement while continuing to work with the ESPCP team from program participation to job retention. At job entry, the treating psychiatrist judged the patient’s mood symptoms to be clinically stable, with no acute suicidality and adequate sleep-wake regularity to begin part-time work. Warning signs for potential relapse were defined pragmatically as sustained insomnia, increased avoidance, and marked irritability or tearfulness lasting several days, based on clinical observations and patient reports.

She secured a one-year fixed-term position in in-house cleaning at approximately 20 hours/week. After job entry, the team provided ongoing consultations focusing on interpersonal stressors and coordination with the employer to adjust support strategies. At follow-up, stress-related maladaptive alcohol use emerged, and the team provided psychoeducation and brief guidance on safer coping strategies and relapse risk management. She maintained employment for 12 months and subsequently transitioned to regular employment at a different company, according to her own preference. During the 12-month follow-up, she took one day of sick leave (for menstrual pain), with no late arrivals or early departures.

The mGAF score improved from 68 at program entry to 78 at program completion. The LASMI domain scores for daily living and work improved (D: 3→2 and W: 6→3), with stable interpersonal relations (I: 10→10) and endurance and stability (E: 2→2) and slight improvement in self-recognition (R: 6→5).

Case 2

A man in his 40s with major depressive disorder resigned from a sales position after symptom exacerbation under increasing performance pressure, resulting in hospitalization and job separation. He participated in two consecutive three-month ESPCP phases (approximately six months). Phase 1 focused on rebuilding confidence through structured daily activities, psychological education, and cognitive-behavioral techniques to identify and reframe negative thoughts. Upon completing phase 1, the patient reported persistently low confidence and requested another phase. Phase 2 strengthened relapse prevention and incorporated individual and family psychoeducation to align self-management strategies with family support.

The patient obtained competitive employment in factory manufacturing. At job entry, the treating psychiatrist judged him to be clinically stable for graded work exposure, with no acute depressive risk and stable adherence to outpatient follow-up. Warning signs were monitored using a brief checklist that was agreed upon by the patient and employer (sleep disruption, fatigue accumulation, increased errors, and withdrawal), prompting additional contact when present. Work hours were increased stepwise (10→15→20→30→40 hours/week) in collaboration with the employer, with approximate durations of one month, 1.5 months, one month, and two months for each stage before reaching 40 hours/week, which has been maintained thereafter. For example, during the 20-hour/week phase, increased fatigue prompted an ad hoc call, leading to a brief adjustment of pacing and scheduled breaks. Regular meetings were conducted to monitor workload, role clarity, and early signs of overextension.

At the two-year follow-up, he continued full-time work (40 hours/week) without taking sick leave. The mGAF score increased from 54 to 61 after phase 1 and to 68 after phase 2. The LASMI domain scores showed stepwise improvements across phases, notably in work (W: 18→11→9) and daily living (D: 7→4→3), with improvements in interpersonal relations (I: 14→14→11), endurance and stability (E: 4→4→3), and self-recognition (R: 6→6→5) by the end of phase 2. He took zero sick leaves, with no late arrivals or early departures during the reported follow-up period.

The Public Employment Security Office helped with job selection and adjusting employment conditions, serving as a bridge at the time of job placement while continuing to work with the ESPCP team from program participation to job retention.

Employer perspectives

For each case, an employer representative participated in a semi-structured interview (approximately 40-60 minutes) discussing the perceived benefits and challenges of collaborating with the hospital team. A brief interview guide covered (i) the perceived value of collaboration; (ii) information needs and confidentiality boundaries; (iii) communication frequency and burden; (iv) workplace adjustments; and (v) recommendations for improvement. The interview data were summarized and organized using inductive content analysis, with codes iteratively refined and grouped into categories [15]. To enhance reporting transparency for qualitative methods, we followed the key items from the COREQ checklist [16]. Given that N = 2, thematic saturation was not assumed, and the findings were presented as context-specific implementation insights.

To protect confidentiality, quotes were paraphrased and de-identified, and employer codes were presented as A (Case 2) and B (Case 1). The resulting themes and representative paraphrased statements are summarized in Table 2. This study was approved by the Ethics Committee of the Faculty of Medicine, Shinshu University (approval numbers 6112 for the patient component and 6184 for the colleague component). Written informed consent was obtained from the patient and all colleague participants.

**Table 2:** Themes from employer interviews with representative statements (de-identified) Code A refers to the employer interviewed for Case 2, and code B refers to the employer interviewed for Case 1. ESPCP = Employment Support Program in Cooperation With the Public Employment Service

Category/theme	Representative statement (paraphrased)	Employer code	Practical implications for ESPCP delivery
Benefits: flexible job design	Task trials and shared observation helped identify what worked and supported flexible adjustment of duties and workload.	B	Use task trials and shared observation to co-design duties and adjust workload proactively.
Benefits: role awareness and consultation window	Having a clear point of contact enabled earlier consultation and reduced the sense of having to handle everything alone.	B	Define a clear point of contact and escalation pathway to reduce burden and enable early consultation.
Benefits: unified support approach	Regular discussions helped align how to respond to problems and what to prioritize.	A/B	Hold brief, repeated triadic discussions to align priorities and response plans across stakeholders.
Benefits: useful information sharing	Sharing the hospital team’s view helped communicate the situation to internal stakeholders; “the hospital perspective was key.”	A	Provide concise, role-specific summaries for employers to relay internally (what to monitor, how to respond, when to consult).
Challenges: communication frequency and burden	Meetings were helpful, but decisions on the right timing and frequency required adjustment.	A/B	Set a default communication rhythm with triggers for ad hoc contact; keep meetings short and agenda-driven.
Challenges: confidentiality versus operational needs	It was sometimes difficult to decide what could be shared to support work while respecting patient privacy.	A/B	Agree in advance on information that could be shared (functional indicators, warning signs, response steps) and document consent boundaries.

## Discussion

Instead of evaluating clinical efficacy, this report clarified practice-relevant mechanisms for employer collaboration within the ESPCP through the practice-oriented integration of two complementary data sources: case-level clinical trajectories and linked employer perspectives. By aligning what was done for each patient with what employers reported as helpful or challenging, we highlighted implementation-relevant mechanisms, such as a clear consultation window, timely information sharing, and repeated triadic discussions, which may support job retention.

From the case trajectories, clinically meaningful changes were most evident in work-related and daily living functioning. The LASMI domain scores suggested clinically meaningful improvement, particularly in work and daily living (Case 1: W: 6→3, D: 3→2; Case 2: W: 18→11→9, D: 7→4→3), consistent with the observed employment retention and graded work-hour expansion. In Case 1, relapse prevention-oriented tailoring included crisis planning, post-hiring interpersonal consultation, and brief guidance on safer coping strategies when stress-related drinking emerged. In Case 2, the structured two-phase WRP approach shifted from confidence rebuilding to strengthened relapse prevention through family psychoeducation, followed by an employer-coordinated stepwise increase in work hours, culminating in sustained full-time employment. The program’s structure aligns with principles emphasized in supported employment and complements the broader literature on Individual Placement and Support [12,13].

Employer perspectives suggested that the effectiveness of collaboration depended on frequent contact and on how communication was structured. Employers valued timely, function-focused information sharing; a clear point of contact; and opportunities for shared problem-solving (Table 2). Simultaneously, they identified confidentiality concerns, stigma, and uncertainty about the communication burden as practical barriers [10,11]. These challenges were most evident when employers noticed increased fatigue, interpersonal strain, or possible early warning signs; however, they were uncertain about what information could appropriately be shared within the workplace and what should remain between the employee and the healthcare team. Therefore, collaboration should be designed around a single consultation window, initially agreed-upon boundaries for sharing employees’ work-relevant functional information, and trigger-based rather than unnecessarily frequent contact, with these boundaries reviewed flexibly as job demands and support needs evolve. Accordingly, confidentiality can be managed while supporting practical workplace decision-making [8,9].

This study had several limitations. First, this report included only two cases from a single setting; therefore, causal inferences could not be drawn. Second, employer perspectives were based on a few interviews and may reflect a selection bias toward more collaborative workplaces. Third, outcomes may depend on local resources (e.g., availability of public employment service officials and day care infrastructure). Future work should evaluate the ESPCP using larger samples, standardized outcomes (e.g., sick leave recurrence [1,2]), and implementation measures while clarifying how confidentiality can be managed to optimize workplace support and collaboration.

## Conclusions

The ESPCP, a psychiatric day care-based employment support model delivered in partnership with the Public Employment Service, supported two patients with major depressive disorder in achieving and sustaining competitive employment. Employer interviews suggested that structured collaboration enables practical workplace adjustments through information sharing, consultation access, and flexible task design. These cases support the feasibility of integrating patient-facing relapse prevention with employer-facing coordination to enhance employment retention.
